# Awareness of Risk Minimization Measures for Valproate and Pregnancy Prevention Program Compliance Among Pharmacists: A Cross-Sectional Survey in Romania

**DOI:** 10.3390/ph18121861

**Published:** 2025-12-05

**Authors:** Madalina Huruba, Daniel Leucuta, Andreea Farcas, Cristina Mogosan

**Affiliations:** 1 Department of Pharmacology, Physiology and Physiopathology, Faculty of Pharmacy, “Iuliu Hațieganu” University of Medicine and Pharmacy, 400349 Cluj-Napoca, Romania; huruba.madalina@umfcluj.ro (M.H.); cmogosan@umfcluj.ro (C.M.); 2 Department of Medical Informatics and Biostatistics, “Iuliu Hațieganu” University of Medicine and Pharmacy, Pasteur Street, No. 6, 400349 Cluj-Napoca, Romania; dleucuta@umfcluj.ro; 3 Pharmacovigilance Research Center, “Iuliu Hațieganu” University of Medicine and Pharmacy, 400349 Cluj-Napoca, Romania

**Keywords:** valproate, risk minimization measures, pregnancy prevention program, pharmacists

## Abstract

**Background/Objectives:** Risk minimization measures, including a pregnancy prevention program (PPP), have been established by the European Medicine Agency to strengthen the restrictions on valproate (VPA) use in pregnant women/women with childbearing potential. We aimed to assess pharmacists’ awareness of the new measures and behavior in terms of compliance to PPP recommendations. **Methods**: We performed a cross-sectional, national, non-interventional survey among pharmacists between December 2024 and February 2025. No sample size calculations were performed. Inclusion criteria were pharmacists active in community pharmacies in Romania. **Results**: In total, 267 pharmacists were included, balanced in terms of age groups, with a slight predominance for 31–40 year olds (33.7%) and mostly female (93.3%). More than half (60.7%) did not recall receiving any type of PPP information (direct healthcare professional communication [DHPC] or educational materials [EMs]). Participants generally read the DHPC, fully (64.1%) or partially (21.4%); all reportedly read the EMs, generally fully (73.0%). Half (145, 54.2%) dispensed VPA at least once during the last 12 months. Among this subgroup, 15.2% used the EMs, 38.6% counseled the patient regarding the VPA teratogenic risk, and 32.4% counseled on the importance of effective contraceptive measures at every VPA dispensing. Neither awareness nor behavior met the pre-established success criteria; therefore, the overall PPP compliance was not demonstrated. **Conclusions**: Despite notable proportions of pharmacists offering counseling when dispensing, overall PPP compliance and EM use could be enhanced. More research is needed to identify why some measures are not properly adhered to, in order to increase the overall risk mitigation efficacy.

## 1. Introduction

Despite sodium valproate (VPA) being regarded as a highly effective antiseizure medication since its first authorization in 1962 [1], its teratogenicity has been recognized for decades as well [2]. The first of such indications date back to the 1980s [3], with recent studies strengthening the association between the use of VPA and teratogenic effects [4,5,6,7]. Major congenital malformations were reported in 6.7% (95% CI: 5.5% to 8.3%) of pregnancies exposed to VPA in utero in a pregnancy registry study conducted in the United Kingdom (UK) and Ireland. Hypospadias and genitourinary tract defects, neural tube, cleft lip and palate, and cardiac defects were among the top ones reported [8]. Moreover, recently published data, including data from the European and International Registry of Antiepileptic Drugs in Pregnancy (EURAP), suggested a high risk of a neurodevelopmental anomaly in children exposed to VPA in utero [7], and dose-dependency [9].

From a pharmacological perspective, VPA teratogenic effects are considered multifactorial, generally involving several overlapping mechanisms. Among these, histone deacetylase inhibition (HDAC) [10,11], folate metabolism disruption [12,13], and oxidative stress [14] are linked with the most pronounced teratogenic effect [15]. Congenital malformation risk tends to be dose-dependent, with the highest noted at doses >1500 mg/day, especially for neural tube defects and cognitive impairments [16]. Higher concentrations of VPA tend to lead to more profound HDAC and folate metabolism inhibition, increased oxidative stress levels in embryonic tissues, and more significant mitochondrial dysfunction, making dose escalation a critical risk amplifier. In theory, extended-release formulations might have the potential to reduce peak embryonic exposure, potentially muting the teratogenic effect at equivalent daily doses. However, regardless of the formulation, the risk cannot be excluded as total daily systemic exposure remains the dominant risk factor [17]. Moreover, even in the context of pregnancy changes in the pharmacokinetics of VPA (e.g., increased volume of distribution, enhanced hepatic metabolism in the second–third trimester, or altered protein binding), free (active) VPA levels may still remain high, especially in high-dose regimens [18]. Alternatively, newer-generation antiepileptic agents such as lamotrigine and levetiracetam demonstrate significantly lower teratogenic risks, with no consistent association with cognitive deficits or major congenital malformations, making them safer options compared to VPA for women with childbearing potential (WCBP). Even carbamazepine, despite implying a moderate risk, particularly at higher doses, still presents a safer profile compared to VPA [5].

To mitigate VPA teratogenic risk, a set of interventions has been established by the European Medicine Agency (EMA) to strengthen the restrictions on the use of VPA in pregnant women or WCBP [19]. Therefore, the use of VPA to treat bipolar disorder, migraine, or epilepsy during pregnancy is contraindicated, and, unless within the pregnancy prevention program (PPP), VPA must not be used by WOCB. However, it is recognized that for some women with epilepsy, stopping VPA treatment might not be possible; therefore, they may have to continue treatment (with appropriate specialist care) during pregnancy [20]. The scope of the program is to ensure that both patients and healthcare professionals (HCPs) are aware of the risks associated with VPA use during pregnancy, as well as to provide guidance for counseling [21]. Nonetheless, balancing the risks and benefits in this context remains a challenge, as oftentimes a good alternative therapy is lacking. Moreover, both legal and ethical considerations need to be taken into account if a woman prefers to continue VPA therapy without any concern for contraception. Overall, the decision to withdraw or continue VPA treatment should be taken based on comprehensive conversations with the patient and by making sure that the risks implied have been well communicated and understood [22].

The PPP includes several activities to be followed, generally aiming to assess the pregnancy potential and navigate the risks associated with VPA use during pregnancy, and the importance of highly effective contraception or actions to be taken in the case of an unplanned pregnancy. Additionally, educational materials (EMs) were designed to improve risk awareness and knowledge regarding the required measures to prevent pregnancies during treatment [21,23].

Our survey was designed to assess the impact of the new, enhanced risk minimization measures (RMMs) on pharmacists. The objectives were to assess pharmacists’ awareness of the new RMMs, and behavior in terms of PPP compliance. Understanding the impact of these RMMs is important in order to ensure the safe use of VPA among WCBP.

## 2. Results

### 2.1. Demographics and General Data (Overall Population)

Our study included a total of 267 participants with valid, complete, and evaluable responses. The participants were generally balanced in terms of age groups, with a slight predominance for 31–40 year olds (33.7%), and most pharmacists were female (93.3%) (Table 1). Approximately half (54.2%) of the participants dispensed VPA at least once during the last 12 months prior to the survey.

All but one participant knew about the teratogenic effects of VPA, with one mentioning they did not know about the risk at all. Regarding the sources researched about the safety concerns of VPA, most participants mentioned that they used the National Agency for Medicines and Medical Devices of Romania (NAMMDR) (59.9%), followed by EMA official websites (24.3%). Notably, 19.5% of the participants mentioned that they did not research any sources at all. Other sources were mentioned by less than 3% of the participants (product information, peer-reviewed journals, courses, etc.).

### 2.2. Communication Receipt and Risk Awareness (Overall Population)

The communication receipt and risk awareness results are presented in Table 2. More than half of the participants (60.7%) did not recall receiving any type of PPP information (direct healthcare professional communication [DHPC] or EMs).

Among those who did received such information, the participants generally reported having read the DHPC, either fully (64.1%) or partially (21.4%). If EMs were received, they were received in print (78.4%), as well as via email (52,7%), and all participants reported that they read the EMs, generally fully (73.0%) (note: conditional on receipt). Of note, despite the majority of participants (86.9%) noticing the QR code embedded on the package, only about half (48.7%) were aware of its purpose; that is, that the code leads to product information and EMs.

**Figure 1:**
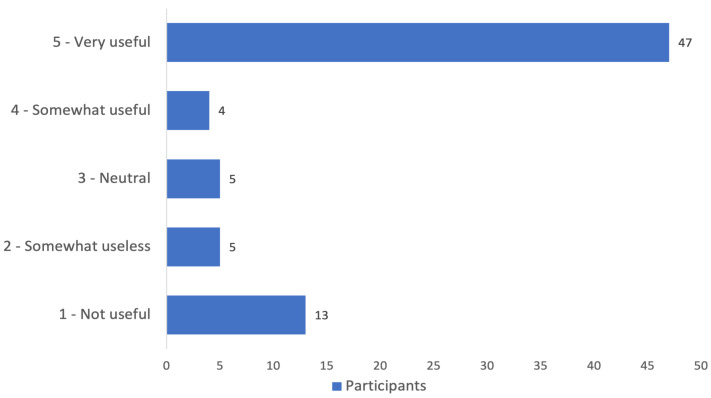
Pharmacists’ impressions of the usefulness of education materials (Likert scale) *. * Figure includes participants who reported receipt of educational materials, *n* = 74.

The pharmacists were generally positive about receiving the EMs (Figure 1), with most (67.6%) finding the HCP guide to be the most useful EM; only 12.2% preferred the patient card.

Subgroup stratification analysis revealed that, generally, the participants aged between 31 and 40 or with work experience between 21 and 30 years tended to have better awareness results (i.e., reading in full, noticed the QR, and had knowledge of its purpose). Similarly, participants who dispensed VPA at least once during the past year tended to score better for these items (see Table S1).

### 2.3. PPP Compliance

#### 2.3.1. Use of EMs

Of note, use of EMs was assessed among all participants who dispensed VPA, not conditional on whether or not they reported receipt. Among the 145 pharmacists who dispensed VPA to WCBP, 15.2% used the EMs at every dispensing, while 61.4% used them only sometimes. When asked about the type of the EM they generally use for patient counseling, the majority (70.3%) mentioned the HCP guide, followed by lower percentages of those who prefer using the patient guide (26.1%), the risk acknowledgment form (17.1%), or patient card (10.8%); other responses (e.g., product information, peer-reviewed sites, or other) were selected by <4% of the participants.

Almost half (47.7%) of the participants (excluding those who never used EMs) reported that they offered the patient card only in some cases; 23.4% participants offered the patient card at every VPA dispense; and 28.8% never offered the card. Among the 34 participants who never used EMs, the justifications varied between the unavailability of the EMs in the pharmacy (79.4%), EMs not being close by, lack of familiarity with EMs (26.5% each), or a lack of notifications from the pharmacy dispensing software (11.8%). These results are presented in Table 3.

**Figure 2:**
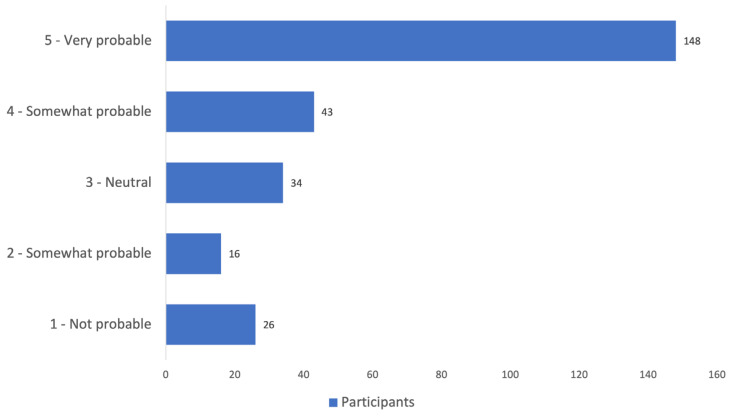
Educational materials or DHPC use in the future (probability Likert scale).

Overall, when asked about the probability of using the EMs or the DHPC in the future, the majority of the participants selected the top two maximum probability scores on the 1 to 5 scale: 55.4%—very probable and 16.1%—somewhat probable (Figure 2).

#### 2.3.2. Counseling

Patient counseling in line with the PPP recommendations was evaluated among the same subgroup of 145 pharmacists who dispensed VPA during the 12 months prior to the study.

The participants generally mentioned that they counsel the patient regarding the teratogenic risk of VPA, most (38.6%) at every VPA dispensing, followed closely by those who only counsel in some cases (36.6%), or when the patient starts a conversation (17.9%); 6.9% of the participants never do. Regarding the importance of effective contraceptive measures, 32.4% participants emphasize it at every VPA dispensing, 35.9% only in some cases, 21.4% if the patient starts a conversation, and 10.3% never counsel the patient on this aspect (Table 3).

When asked about their approach towards a patient with an unplanned pregnancy during VPA treatment, the majority of the participants (88.3%) mentioned that they would advise patients to urgently consult their prescribing physician, 41.4% would counsel the patient regarding the teratogenic risk associated with VPA use during pregnancy, and 23.3% would advise their patients to continue treatment until the next visit to a physician.

Other recommendations pharmacists offered during patient counseling included reminding the patient about the need to periodically evaluate their treatment (at least annually) (73.1%), referring the patient to their treating physician because they were not using effective contraception (40.0%), and advising the patient to access the QR code embedded on the secondary package of VPA medicinal products (19.3%).

When asked what they consider would facilitate patient counseling and PPP implementation at the community pharmacy level in Romania, the response options were balanced between availability of the printed EMs in the pharmacy (27.8%), training on EMs’ information and their use as well as the importance of patient counseling (26.4%), remuneration for counseling (22.2%), or more time available for patient counseling (18.8%).

The majority of the participants (82.8%) reported that they generally do open the VPA package in order to partially dispense the medicine. Among them, only a third (37.5%) included a copy of the patient leaflet or patient card in these situations (Table 3).

Participants aged between 31 and 40 years generally had better results, whereas work experience did not seem to impact the PPP compliance results (see Table S2).

#### 2.3.3. PPP Success Evaluation

PPP compliance at the individual level revealed success for a total of 52/267 and 31/145 pharmacists for the awareness and behavior dimension, respectively, which is 26.7% and 21.4% of the total pharmacists evaluated for each dimension, respectively. Neither dimensions met the success criteria; therefore, the overall PPP compliance was not demonstrated.

## 3. Discussion

This is the first study to evaluate pharmacists’ awareness of the RMMs for VPA use during pregnancy and PPP compliance in Romania. Similar cross-sectional surveys have been conducted as part of the multinational post-authorization safety studies (PASS) in France, the UK, Sweden, Poland, Germany, and Spain, targeting both HCPs and patients [24,25]. Other similar, national studies have been conducted in Latvia [26], Belgium [27], and Ireland [28]. Objectives such as risk awareness and knowledge evaluation, as well as behavior from clinical and pharmaceutical practice in close correlation with the RMMs and PPP, were evaluated in these studies. A recurrent annual pharmacist survey has been conducted in France since 2016, per the requirements of the national health authority [25]. Notably, when strictly referring to the number of pharmacists included in each of these countries, our analysis generally included a higher number of pharmacists, even after excluding those who did not dispense VPA within 12 months before the survey (145 vs. between 25 and 98 participants in other similar studies) [21,25,26].

In our analysis, approximately half of the participants reported having dispensed VPAs to WCBP during the 12 months prior to survey participation. Despite this being a notable finding, whether or not pharmacists dispensed VPAs to WCBP did not represent an exclusion criterion, as risk awareness and knowledge measures targeted all pharmacists (alongside other HCPs), not conditional on whether or not they generally dispense VPAs [19]. This particular survey question was designed to offer an overview of the frequency of VPA dispensing among the study participants. That being said, PPP compliance with regard to counseling was assessed in a subgroup comprising participants who reportedly did dispense VPA within the timeframe of interest.

Overall, less than half of the participants (39%) in our analysis actively received any type of information regarding the PPP, and all but one were aware of the associated teratogenic risk. Colas et al. reported slightly higher levels of receipt of EMs, ranging from 27% to 66% per type of EM (overall group of pharmacists from France, the UK, Sweden, Poland, Germany, and Spain) [25], while other studies reported that 78.6% of the pharmacists in Denmark and 82.3% of community pharmacists in Ireland reported receipt of materials [28]. Our analysis awareness (defined as receipt and reading of relevant EMs) results tended to be in line with those of Colas et al., where 34.0% of pharmacists included in the six European countries reported awareness [25]. In our study, most participants were aware of the risk for more than six years prior to the study. In Denmark, 51% of pharmacists had heard about the teratogenic effects in the past 5 years [21]. In Latvia, pharmacists had only more recently become aware of the associated teratogenicity (47.9% within the past two years) [26].

The most common information sources for pharmacists in our study were health authorities’ official websites, in line with other results in the literature [26,28]. As these sources are known for reporting valid, up-to-date information and include the latest regulatory recommendations, this can be regarded as a positive aspect. Notably, almost a quarter of the participants reported that they did not check any sources related to VPA safety concerns. In the context of the importance of the teratogenic risk, the constant risk mitigation measures, and low rate of receipt of EMs/DHPC, this aspect may be viewed as concerning. Granted, the teratogenic risk associated with VPA use during pregnancy is not new information; therefore, HCPs might consider these data to already be known. Even so, researching sources such as regulatory official websites and/or materials specifically designed to be disseminated is encouraged in order to remain informed in the context of new restrictions of use.

The participants in our analysis generally read (most in full) the EMs/DHPC received, in line with the multinational European survey conducted by Colas et al. [25]. The most frequently received EM was the risk acknowledgment form (41.9%) and patient guide (39.2%). The patient card scored the lowest, with only 13.5% of the participants recalling having received it, despite this generally being embedded on the secondary package of VPA medicinal products per EMA recommendations [19], in addition to the embedded QR code leading to the EMs, including the patient card [29]. In theory, the patient card should be the most accessible EM, yet it scored lowest on all the survey questions, probably also because it addresses patients primarily. Generally, the participants in our study tended to have a positive attitude towards utilizing EMs, in line with the study conducted in Ireland, reporting high awareness and use of EMs amongst community pharmacists [28]. In our study, the majority of participants found EMs very useful, with the HCP guide and risk acknowledgment form being the most preferred. Predictably, the patient card scored the lowest on the utility Likert scale, with only 12.2% of participants preferring to it. In Denmark, the patient reminder card and the HCP guide were the least used EMs [21]. In Latvia, among pharmacists, the HCP guide, pharmacist checklist (country specific), and patient card were the least recognizable EMs [26].

As mentioned above, per the updated text of product information post-EMA safety evaluation, a QR code should be included in the package design and/or patient information leaflet. Scanning this code should lead to updated detailed product information [29]. Despite the vast majority of participants mentioning that they were aware of the QR code, in the sense that they did see it, only about a half knew that it should lead to the EMs, including the patient card. These results, in line with the generally low scores achieved for the patient card, could indicate a poor communication of the updates to product information, particularly those of the secondary package design. That might be viewed as unfortunate, as through its accessibility, this could represent a valuable resource, which, based on the results, seems to be underutilized. Moreover, counseling on this aspect would be beneficial as patients should be encouraged to access the materials that are available and specifically designed for them.

When asked about the frequency of using the EMs, in our study, only 15.2% of the participants who dispensed VPAs were compliant with the PPP requirements, i.e., they used EMs at every VPA dispensing; 23.4% of the participants, however, reportedly never use the EMs when dispensing VPA. By far the top most preferred EM was the HCP guide, unlike participants in Denmark, where the HCP guide was among the least preferred EM [21]. In our study, the patient card scored the lowest, with only 10.8% of participants reporting its use during counseling. When asked about offering the patient card and counseling based on it, only about a quarter reported that they offer it and counsel based on it at every VPA dispensing and thus were compliant with the PPP. A slightly higher percentage of community pharmacists (17.7%) in Ireland reported that they provide the patient card every time VPA is dispensed [28], while Oliveri et al. reported an even lower percentage of pharmacists in Denmark (compared to those in Romania) who used the patient card (5.1%) [21]. Better scores for this variable have been obtained by Colas et al., where 72% of the pharmacists from the six European countries reportedly offered the patient card every time VPA was dispensed or to new patients [25]. In the context of the patient card generally being embedded on the secondary package for most of the products available on the market (per EMA requirements [19]), one could attest that the patient card is in fact offered at every VPA dispensing, except for partial dispensing. Therefore, these results may be perceived as more of an indicator of the awareness/information related to this particular EM as opposed to pharmacy practice (i.e., compliance with PPP).

In the case of partial dispensing, only a third of the participants in our study reported that they include a copy of the patient information leaflet or patient card. Better scores have been reported in Ireland, where more than half of the community pharmacists reported that they provide a copy of the package leaflet to the patient in case of partial dispensing [28].

When asked about the reasons why they do not use EMs, the most frequent response was these not being available in the pharmacy (79.4%), followed by not having them close by (38.2%), and a lack of familiarity with EMs (26.5%). A lower proportion (11.8%) selected the lack of electronic system notification when dispensing VPA. The first argument could be supported by the reduced receipt levels (overall, less than half of the participants recalled receiving any type of EMs). Moreover, the preferred dissemination channel was via electronic means, whereas for patient counseling, printed materials would have probably been handier. The second argument, the lack of familiarity with the EMs, was also part of Colas et al.’s results, where participants lacked familiarity and knowledge with respect to the key information enclosed [25]. Similar barriers were reported in the literature, such as a lack of materials [26], lack of familiarity/insufficient knowledge of the PPP, lack of time [25,26], or insufficient integration of these measures into the daily workflow [25]. Furthermore, in Denmark, the topic of pregnancy was often seen as a private matter, potentially unsuitable to discuss at the pharmacy counter [21]. As improvements, pharmacists in our study mentioned the availability of the EMs in the pharmacy, courses or training on using the EMs and the importance of counseling patients using VPA, more time available to dedicate to patient counseling, or service remuneration. Pharmacists in Demark stated the preference for more campaigns to highlight the important information about the PPP in relation to VPA dispensing as well, in addition to the implementation of a warning in the electronic dispensing system [21]. Counseling remuneration and time available for patient counseling in the pharmacy in Romania are aspects already debated in the literature [30], and, as such, these might justify part of the pharmacists’ responses. Providentially, the availability of printed EMs and informing or training pharmacists are in fact accessible and feasible to implement. The latter could be taken into account when/if improving the implementation of RMMs in Romania. Other potential pragmatic improvement suggestions at the pharmacy level could include displaying a QR code poster at the counter linking to VPA safety information and EMs, or adopting a concise counseling script emphasizing key messages for patients (i.e., contraception, treatment review, documentation). At the system level, the integration of e-dispensing alerts when VPA is scanned for WCBP and the availability of printed EMs (patient card, checklist, and poster) close by would be helpful. Ultimately, these results could indicate that there is space for improvement when it comes to process implementation and pharmacist training in Romania.

When asked about the likelihood of using EMs or the DHPC to aid patient counseling in the future, most pharmacists selected the top probability on the Likert scale (very probable, 55%; somewhat probable, 16%), suggesting a potential positive impact of conducting such surveys through highlighting the importance of such actions. A similar positive impact on the future use of EMs was reported in Latvia [26] as well as Denmark (but on the prescribers) [21].

In terms of patient counseling, less than half of the pharmacists in our study counseled the patient regarding the associated teratogenic risk (38.6%), or emphasized the importance of effective contraception (32.4%) at every VPA dispensing. Better behavior scores were noted in Denmark [21] and Latvia [26], where pharmacists most often provided information about the importance of effective contraception and advised patients to contact their prescriber if pregnancy was suspected. Despite being notably reduced comparatively, in the context of the PPP, the fact that some pharmacists in our study never counseled the patient regarding the teratogenic risk or need for contraception during VPA treatment (7% and 9%, respectively) may be viewed as concerning. Per the PPP recommendations [19], such counseling should be offered at every VPA dispensing or to new patients. When asked about the approach towards a patient with an unplanned pregnancy during VPA treatment, the majority of pharmacists (84%) in our study mentioned that they would immediately refer the patient to the treating physician overseeing her treatment; lower proportions would counsel the patient on the teratogenic risk (41.4%) or on the importance of not stopping VPA therapy unless under doctor supervision (23.4%). Similar results were reported in Denmark, where the majority of pharmacists never or seldom advised patients to stop taking VPA if they suspected a woman to be pregnant [21]. On the other hand, Colas et al. reported better scores for the similar question included in their survey across Europe, with more than 70% of pharmacists offering all three recommendations in the abovementioned scenario [25]. Other recommendations offered by the pharmacists in our study generally included reminding the patient about the need to reevaluate the treatment at least annually (73.1%), or referring the patient to their general practician as they were not using effective contraception (40.0%). Similar results have been reported by Colas et al., noting a 49.7% success rate for behavior questions among pharmacists [25].

Overall, when comparing our results with other studies conducted in European countries (see Table S3), we observed that the implementation of these measures remains inconsistent across community pharmacies. Similarly to other European surveys’ findings, our Romanian sample showed relatively low levels of routine counseling, patient card provision, and reinforcement of contraception during dispensing, in contrast with high adherence in scenarios involving unplanned pregnancy. Receipt of EMs was also variable across studies, with our survey identifying one of the lowest levels of active PPP information dissemination. Implementation of the VPA PPP in community pharmacies seems to be limited by low material availability, inconsistent counseling, and gaps in the routine dispensing of EMs. Overall, the gaps observed in our study seem to be in line with internationally reported challenges and highlight the need for more structured support to strengthen PPP implementation in pharmaceutical practice.

The PPP compliance success evaluation for the two main dimensions targeted by our survey (i.e., awareness and behavior) revealed low success rates for both, strengthening the observed gaps in PPP implementation in Romania.

### Strengths and Limitations

Cross-sectional surveys have the advantage of being relatively fast and cost-effective. Moreover, such approaches are valuable for generating hypotheses, and the results of such studies can form the base for future extended research in the field, including qualitative research in order to better understand the implementation of RMMs in clinical practice.

A well-known limitation of surveys is their low response rate. Our survey was distributed via professional association mailing lists, as well as via social media. In light of the latter, as well as accounting for social media users disseminating the link to colleagues, a thorough estimation of the number of pharmacists targeted is not possible, including response rates based on this. National-level population margins for pharmacists were not obtainable at the time of this study. Our sample constitutes a non-probability convenience sample, and the findings should be interpreted with caution regarding generalizability. Nevertheless, in our analysis, the number of respondents was comparable with those reported in similar studies in the literature. To ensure participant anonymity, pharmacy identifiers were not collected; therefore, we were unable to model potential clustering of respondents within the same pharmacy. If such clustering was present, standard errors may be underestimated and CIs correspondingly narrower. This design effect should be considered when interpreting the precision of the estimates.

The variability in the responses based on the participants’ time since awareness introducing potential recall bias is a well-known limitation in survey research, particularly for retrospective studies. Participants may struggle to accurately remember past events or experiences, potentially leading to inaccurate or incomplete reporting. We aimed to reduce recall bias by using a relatively short recall period (i.e., 12 months) to enhance the accuracy and consistency of the participants’ reports.

Respondent bias is also noted as a limitation of retrospective surveys, as participants might have the tendency to not respond honestly or even misinterpret data due to social desirability or misunderstanding. Moreover, social desirability bias may have influenced responses to counseling-related items, with participants potentially overstating their counseling practices, as well as non-response bias as pharmacists who participated may differ from those who did not. To mitigate these limitations, we employed careful question phrasing and referred to other similar data sources. Nevertheless, surveys remain a valuable and efficient tool for gathering data. Future studies should seek to validate self-reported counseling behaviors using objective data sources, such as dispensing-system logs or direct pharmacy audits, to strengthen the accuracy and reliability of the findings.

## 4. Materials and Methods

### 4.1. Study Design

This was a cross-sectional, national, non-interventional survey conducted among pharmacists, part of the key contributors in risk minimization strategies through patient counseling. The survey was conducted in Romania from 23 December 2024 to 28 February 2025. The online survey (see Table S4) was disseminated via social media (in particular via social groups including pharmacists) and local pharmacist organization mailing lists; the survey was anonymous and targeted pharmacists who provided their consent to participate. Because social media dissemination prevents a reliable denominator, a formal response rate could not be computed. Specific confirmation of their status as pharmacists working in community pharmacies was required; however, no specific pharmacy identifying details were collected, thus ensuring pharmacy anonymity. The survey remained open for approximately two months, with the initial dissemination on 23 December 2024, and the final reminder sent on 11 February 2025. Two additional reminders were distributed during the month of January.

No sample size calculations were performed. Inclusion criteria were pharmacists active in community pharmacies in Romania.

### 4.2. Survey Methodology

Similar questionnaires used to assess the impact of regulatory agencies’ recommendations on risk awareness and knowledge (targeting both patients and HCPs), prescribing behavior, or adherence to RMMs (addressed to HCPs) have been identified in the literature [31,32]. These sources were used as references in the development of our questionnaire, which was adapted afterwards to the objectives of the present study in the context of risk communication in Romania. The recent Reporting Recommendations Intended for Pharmaceutical Risk Minimization Evaluation Studies: Standards for Reporting of Implementation Studies Extension (RIMES-SE) were used to guide the development of the survey, in line with GVP Module XVI recommendations (see Table S5) [33,34]. Our questionnaire was specifically designed to target pharmacists and included closed- and open-ended questions, either mandatory or optional. In some cases, participants were redirected to specific relevant questions dependent on their responses (e.g., participants who reported they did not receive PPP information automatically skipped questions further inquiring on channels or reading of the materials). Survey questions were designed to evaluate demographic data (age, gender, years of professional experience, and work setting), the level of awareness regarding the new restrictions (13 questions), and pharmacists’ behavior, particularly their compliance with the RMM requirements (10 questions).

The use of EMs was a key area of interest in our study; therefore, the perceived usefulness of these materials was assessed using a Likert scale from 1 to 5, where 1 indicated “not useful at all” and 5 indicated “very useful”. Similarly, the likelihood of using EMs in the future was evaluated, where 1 meant “not likely at all” and 5 “very likely”. For variables measuring frequency of EM use, the following response options were generally available: “only in some cases”, “at each dispensing”, “only if the patient initiates a conversation”, or “never”. The open-ended questions asking pharmacists for their opinions on the practical implementation of RMMs were optional and served as an exploratory objective of the study (3 questions).

A questionnaire was considered evaluable if it included valid and assessable responses to all questions marked as mandatory. The results were analyzed descriptively and stratified according to the participants’ characteristics (subgroup analyses were performed by years in practice, dispensing (yes/no) and age).

With respect to RMMs’ impact, two dimensions were evaluated, awareness (the overall population) and behavior related to measures of the PPP (i.e., compliance, evaluated in the subgroup of participants who dispensed VPA in the previous 12 months at least once). The awareness was evaluated based on the percentage of pharmacists who reported having received and read the relevant EMs. For the behavior dimension, we evaluated the proportion of pharmacists who reported compliance to PPP recommendations.

### 4.3. Statistical Methods

All analyses were primarily descriptive in nature—that is, no comparisons or regression modeling were conducted—and the findings are descriptive and hypothesis-generating only. Percentages were computed with 95% CIs for relevant outcomes.

For PPP compliance, we evaluated the success of the two dimensions (i.e., awareness and behavior in terms of counseling) both at the individual and overall levels. Specifically, we mapped selected survey items related to the two dimensions as well as related answers, compiling a minimum set necessary to prove PPP compliance, taking into account the national DHPC recommendations for pharmacists. The assessment of awareness was based on the percentage of pharmacists who answered that they had received and read (in full) the DHPC and EMs. Behavior (counseling) scores were assessed as the proportion of pharmacists with correct answers to relevant questions (Table S6). PPP compliance was evaluated and considered successful (1) at the individual level if ≥80% of the items for that dimension were correctly answered and (2) at the overall level if the proportion of successful pharmacists was ≥90% for both dimensions, similar to a previous survey on VPA PPP adherence conducted in six EU countries and taking into account the PRAC recommendations [25].

## 5. Conclusions

Less than half of the participants recalled having received any type of information on the PPP. Generally, the patient card was the least appreciated and used EM.

Despite notable proportions of participants offering counseling when dispensing (regarding VPA teratogenic risk, the importance of effective contraceptive measures, or proper management of an unplanned pregnancy), overall pharmacists’ compliance and EM use could be enhanced. In that sense, more research is needed to identify the reasons why some measures are not properly adhered to in order to increase the overall risk mitigation efficacy.

Overall, when asked about the probability of using the EMs or the DHPC in the future, the majority of the participants were likely or very likely to, ultimately suggesting a positive impact of conducting such studies.

## Figures and Tables

**Table 1:** Demographics and general data (*n*, 267 participants).

Demographic Criteria	Participants *n* (% [95% CI])		Participants *n* (% [95% CI])
Age group (years)		Frequency dispensing VPA over the past 12 months	
≤30	77 (28.8 [23.6–34.7])	Once or twice per week	18 (6.7 [4.2–10.6])
31–40	90 (33.7 [28.1–39.8])	Twice per month	18 (6.7 [4.2–10.6])
41–50	73 (27.3 [22.2–33.2])	Once per month or less	109 (40.8 [34.9–47])
>50	27 (10.1 [6.9–14.5])	Never	122 (45.7 [39.6–51.9])
Sex		Timeframe for VPA teratogenic risk awareness ^1^	
Female	249 (93.3 [89.4–95.8])	During the past 2 years	42 (15.7 [11.7–20.9])
Male	18 (6.7 [4.2–10.6])	During the past 6 years	72 (27.0 [21.9–32.9])
Work experience (years)		More than 6 years before the study	139 (52.1 [46.1–58.4])
0–5	66 (24.7 [19.8–30.4])	Other timeframes (free text)	13 (4.9 [2.7–8.4])
6–10	68 (25.5 [20.4–31.2])	Scientific sources researched ^2^	
11–20	66 (24.7 [19.8–30.4])	NAMMDR website	160 (59.9 [53.8–65.8])
21–30	57 (21.3 [16.7–26.9])	EMA website	65 (24.3 [19.4–30])
>30	10 (3.7 [1.9–7])	NA	52 (19.5 [15–24.8])
Region			
Urban	245 (91.8 [87.6–94.6])		
Rural	22 (8.2 [5.4–12.4])		

NAMMDR, National Agency for Medicines and Medical Devices of Romania; EMA, European Medicine Agency; NA, not applicable; VPA, valproate(s); CI, confidence interval; n, number of participants. ^1^ One (1) participant mentioned they did not know about the risk. ^2^ Other sources were researched by less than 3% of the participants. More responses were possible per participant.

**Table 2:** Communication receipt and risk awareness.

Criteria	Participants n (% [95% CI])	Criteria	Participants n (% [95% CI])
Receipt of information on VPA PPP (267 responses)		Channel for receipt of EMs (74 responses)	
Yes	105 (39.3 [33.5–45.5])	Printed	59 (78.4 [68.5–87.8])
No	162 (60.7 [54.5–66.5])	Via e-mail	39 (52.7 [40.8–64.3])
Receipt of DHPC (105 responses)		Reading of the EMs (74 responses) ^†^	
Yes, via e-mail	45 (42.9 [33.4–52.9])	Yes, in full	54 (73 [61.2–82.3])
Yes, printed	42 (40.0 [30.7–50])	Yes, partially	20 (27 [17.7–38.8])
No	18 (17.1 [10.7–26])	No	-
Reading of the DHPC (103 responses) ^†^		Most useful EMs for patient counseling (74 responses *)	
Yes, in full	65, (63.1 [53.0–72.1])	Patient guide	32 (43.2 [31.9–55.2])
Yes, partially	22 (21.4 [14.1–30.8])	Patient reminder card	9 (12.2 [6.1–22.3])
No	15 (14.6 [8.6–23.2])	Risk acknowledgment form	25 (33.8 [23.5–45.8])
Receipt of EMs on safety of VPA during pregnancy (105 responses)		HCP guide	50 (67.6 [55.6–77.7])
Yes	74 (70.5 [60.7–78.8])	Noticing the QR code on the secondary package (267 responses)	
No	31 (29.5 [21.2–39.3])	Yes	232 (86.9 [82.1–90.6])
Type of EMs received (74 responses)		No	35 (13.1 [9.4–17.9])
Patient guide	29 (39.2 [28.3–51.3])	Awareness of QR code leading to drug safety information and EMs (267 responses)	
Patient card	10 (13.5 [7–23.9])	Yes	130 (48.7 [42.6–54.8])
Risk acknowledgment form	31 (41.9 [30.7–53.9])	No	137 (51.3 [45.2–57.4])
HCP guide	60 (81.1 [70–88.9])		

DHPC, direct healthcare professional communication; EMs, educational materials; HCP, healthcare professionals; QR, quick response; VPA, valproate; CI, confidence interval. * More than one response option could be selected. ^†^ Reading of materials (i.e., DHPC or EMs) was conditional on receipt.

**Table 3:** Pharmacists’ adherence to the valproate pregnancy prevention program (subgroup, 145 participants who dispensed valproates 12 months prior to the study).

PPP Adherence Criteria (n, 145 Participants Who Dispensed VPA Within the Past 12 Months)	Participants n (% [95% CI])
**Use of EMs**	
How often do you use the EMs when dispensing VPA? * (n, 145 responses)	
At every dispensing	22 (15.2 [10–22.3])
Only sometimes	89 (61.4 [52.9–69.2])
Never	34 (23.4 [17–31.3])
What type of EMs do you use when counseling patients using VPA (111 responses, excluding participants who never use EMs, n = 34)	
Patient guide	29 (26.1 [18.5–35.5])
Patient card	12 (10.8 [6–18.5])
Risk acknowledgment form	19 (17.1 [10.9–25.7])
HCP guide	78 (70.3 [60.7–78.4])
Other	<4%
How often do you offer the patient card and counsel using it when dispensing VPA? (111 responses, excluding participants who never use EMs, n = 34) *	
At every dispensing	26 (23.4 [16.1–32.6])
Only sometimes	53 (47.7 [38.3–57.4])
Never	32 (28.8 [20.8–38.3])
What is the reason behind not using EMs for counseling patients using VPA? (34 responses)	
Not available in pharmacy	27 (79.4 [61.6–90.7])
Not familiar with the EMs	9 (26.5 [13.5–44.7])
Do not have the EMs at hand	13 (38.2 [22.7–56.4])
Not notified by the pharmacy dispensing system	4 (11.8 [3.8–28.4])
**Patient counseling**	
How often do you counsel the patient on the teratogenic risk associated with VPA use during pregnancy? * (n, 145 responses)	
At every dispensing	56 (38.6 [30.8–47.1])
Only sometimes	53 (36.6 [28.8–45])
Only if the patient initiates a conversation	26 (17.9 [12.2–25.4])
Never	10 (6.9 [3.5–12.7])
How often do you emphasize the importance of effective contraception? * (n, 145 responses)	
At every dispensing	47 (32.4 [25–40.8])
Only sometimes	52 (35.9 [28.2–44.3])
Only if the patient initiates a conversation	31 (21.4 [15.2–29.1])
Never	15 (10.3 [6.1–16.8])
How do you proceed if a patient undergoing VPA treatment has an unplanned pregnancy? (n, 145 responses)	
Advise the patient to urgently consult their prescribing physician	128 (88.3 [81.6–92.8])
Counsel the patient regarding the teratogenic risk associated with VPA use during pregnancy	60 (41.4 [33.4–49.9])
Counsel the patient to continue treatment until the next visit to a physician	34 (23.4 [17–31.3])
Other recommendations offered during patient counseling (n, 145 responses)	
Remind the patient about the need to periodically evaluate their treatment (at least annually)	106 (73.1 [65–80])
Refer the patient to their treating physician because they were not using effective contraception	58 (40.0 [32.1–48.5])
Counsel the patient to access the QR code embedded on the secondary package of VPA medicinal products	28 (19.3 [13.4–26.9])
Other	2 (1.4 [0.2–5.4])
What do you think would facilitate patient counseling and PPP adherence when it comes to pharmaceutical practice in Romania? (n, 144 responses, optional)	
Availability of EMs in pharmacy	40 (27.8 [20.8–36])
More time available to dedicate to patient counseling	27 (18.8 [12.9–26.3])
Courses or training on using the EMs and the importance of counseling patients using VPA	38 (26.4 [19.6–34.5])
Remuneration of counseling	32 (22.2 [15.9–30.1])
**Partial dispensing**	
Do you generally open the secondary package to partially dispense the medicine? * (n, 145 responses)	
Yes	120 (82.8 [75.4–88.3])
No	25 (17.2 [11.7–24.6])
Do you offer the patient card or a copy of the patient information leaflet when partial dispensing is employed? (120 responses, excluding participants who did not report partial dispensing, n = 25) *	
Yes	45 (37.5 [29–46.8])
No	75 (62.5 [53.2–71])

EMs, educational materials; HCP, healthcare professionals; NA, not applicable; PPP, pregnancy prevention program; QR, quick response; VPA, valproate; CI, confidence interval. * Question restricted to one response option.

## Data Availability

Data are contained within the article.

## Supplementary Materials

The following supporting information can be downloaded at: https://www.mdpi.com/article/10.3390/ph18121861/s1, Table S1: Work experience and frequency of dispensing stratification; Table S2: Age group and work experience stratification—PPP compliance; Table S3: Benchmark of VPA PPP implementation at community pharmacy level; Table S4: Questionnaire (EN translation); Table S5: Reporting recommendations Intended for pharmaceutical risk Minimization Evaluation Studies-Standards for Reporting Implementaon Studies (StaRI) Extension: The RIMES-SE Checklist; Table S6: PPP compliance items for pharmacists.

## Abbreviations

The following abbreviations are used in this manuscript:

CI	Confidence interval
DHPC	Direct healthcare professional communication
EM	Educational material
EMA	European Medicine Agency
EURAP	European and international registry of Antiepileptic drugs in Pregnancy
HCPs	Healthcare professionals
MDPI	Multidisciplinary Digital Publishing Institute
NAMMDR	National Agency for Medicines and Medical Devices of Romania
PPP	Pregnancy prevention program
QR	Quick response
RMM	Risk minimization measure
UK	United Kingdom
VPA	Valproate
WCBP	Women with childbearing potential
