# Supplementary material for: Awareness of Risk Minimization Measures for Valproate and Pregnancy Prevention Program Compliance Among Pharmacists: A Cross-Sectional Survey in Romania
Source: Pharmaceuticals (Basel). 2025 Dec 5;18(12):1861. doi: 10.3390/ph18121861 (PMC12735724; doi:10.3390/ph18121861)
Supplement: Supplementary file 1 [file pharmaceuticals-18-01861-s001.zip › SupplMat01_Subgroup_12nov25.pdf]

Supplementary Material 1

Table S1 **Work experience and frequency of dispensing stratification**

												**Total**
	Age group (years)				***Work experience (years)***					**Dispensing (Y/N)**		
	≤30	***31-40***	***41-50***	***>50***	***0-5***	***6-10***	***11-20***	***21-30***	***>30***	**Y**	**N**	
Participants, N	***77***	***27***	***90***	***27***	***66***	***68***	***66***	***57***	***10***	***145***	***122***	***267***
	Reading of the DHPC [n (%); total, 103 responses]											
Yes, fully	*9*	*19*	*2*	*11*	*6*	*16*	*19*	*20*	*5*	37	29	66 (64.1)
Yes, partially	*4*	*8*	*9*	*1*	*3*	*5*	*6*	*8*	-	13	9	22 (21.4)
No	*3*	*9*	*2*	*1*	*4*	*4*	*6*	*1*	-	9	6	15 (14.6)
Total	*16*	*36*	*38*	*13*	*16*	*25*	*31*	*29*	*5*	59	44	103
	Reading of the EMs [n (%); total, 74 responses]											
Yes, fully	*9*	*14*	*22*	*9*	*6*	*12*	*16*	*16*	*4*	30	24	54 (73.0)
Yes, partially	*1*	*10*	*7*	*2*	*1*	*6*	*6*	*6*	*1*	11	9	20 (27.0)
No	-	-	-	-	-	-	-	-	-	-	20	-
Total	*10*	*24*	*29*	*11*	*7*	*18*	*22*	*2*	*5*	41	33	74
	**Noticed the QR code on the secondary package [n (%); total, 267 responses]**											
Yes	*63*	*80*	*64*	*25*	*53*	*61*	*56*	*54*	*8*	135	97	232 (86.9)
No	*14*	*10*	*9*	*2*	*13*	*7*	*10*	*3*	*2*	10	25	35 (13.1)
Total	*77*	*90*	*73*	*27*	*66*	*68*	*66*	*57*	*10*	145	122	267
	**Awareness of QR code leading to drug safety information and EMs [n (%); total, 267 responses]**											
Yes	*32*	*46*	*36*	*16*	*21*	*40*	*32*	*30*	*7*	**74**	**56**	130 (48.7)
No	*45*	*44*	*37*	*11*	*45*	*28*	*34*	*27*	*3*	**71**	**66**	137 (51.3)
Total	*77*	*90*	*73*	*27*	*66*	*68*	*66*	*57*	*10*	**145**	**122**	267

Abbreviations: EMs, educational materials; n, number of participants; QR, quick response; VPA, valproate;

Table S2 **Age group and work experience stratification – PPP compliance**

	**PPP ADHERENCE CRITERIA (n, 145 participants who dispensed VPA during the past 12 months)**									**Total**
	Age group (years)				***Work experience (years)***					
	≤30	***31-40***	***41-50***	***>50***	***0-5***	***6-10***	***11-20***	***21-30***	***>30***	
	**USE OF EMs**									
	How often do you use the EMs when dispensing VPA? Total, 145 participants who dispenced VPA during the past 12 months*									
Participants, N	***45***	***49***	***35***	***16***	***36***	***39***	***35***	***30***	***5***	**145**
At every dispensing	7	4	8	3	5	5	6	5	1	22 (15.2)
Only sometimes	26	31	22	10	23	23	18	23	2	89 (61.4)
Never	12	14	5	3	8	11	11	2	2	34 (23.4)

	What type of EMs do you use during counseling patient using VPA Total, 111 responses – excluding the 34 participants who never use EMs									
Participants, N	***33***	***35***	***30***	***13***	***28***	***28***	***24***	***28***	***3***	**111**
Patient guide	6	9	10	4	5	10	6	8	-	29 (26.1)
Patient card	6	2	4	-	5	2	3	2	-	12 (10.8)
Risk acknowledgment form	3	5	8	3	2	4	5	6	2	19 (17.1)
HCP guide	24	27	19	8	19	21	18	18	2	78 (70.3)
	*How often do you offer the patient card and counsel on the same when dispensing VPA? Total, 111 responses – excluding the 34 participants who never use EMs**									
Participants, N	***33***	***35***	***30***	***13***	***28***	***28***	***24***	***28***	***3***	**111**
At every dispensing	7	5	9	5	6	3	8	8	1	26 (23.4)
Only sometimes	13	21	16	3	10	19	11	12	1	53 (47.7)
Never	13	9	5	5	12	6	5	8	1	32 (28.8)
	What is the reason behind not using EMs for counseling patients using VPA? Total, 34 responses who never use EMs									
Participants, N	***12***	***14***	***5***	***3***	***8***	***11***	***11***	***2***	***2***	**34**
Not available in pharmacy	10	10	5	2	6	9	9	1	2	27 (79.4)
Not familiar with the EMs	5	4	-	-	3	4	2	-	-	9 (26.5)
Do not have the EMs at hand	7	4	1	1	4	5	3	1	-	13 (38.2)
Not notified by the pharmacy dispensing system	1	2	1	-	1	2	1	-	-	4 (11.8)
	PATIENT COUNSELING									
	How often do you counsel the patient on the teratogenic risk associated with VPA use during pregnancy? Total, 145 participants who dispenced VPA during the past 12 months*									
Participants, N	***45***	***49***	***35***	***16***	***36***	***39***	***35***	***30***	***5***	**145**
At every dispensing	14	18	16	8	10	15	15	14	2	56 (38.6)
Only sometimes	15	19	14	5	13	13	14	11	2	53 (36.6)
Only if the patient initiates a conversation	10	10	4	2	10	6	5	4	1	26 (17.9)
Never	6	49	35	1	3	5	1	1	-	10 (6.9)
	How often do you emphasize the importance of effective contraception? Total, 145 participants who dispenced VPA during the past 12 months*									

Participants, N	***45***	***49***	***35***	***16***	***36***	***39***	***35***	***30***	***5***	**145**
At every dispensing	11	15	14	7	8	14	10	13	2	47 (32.4)
Only sometimes	14	19	13	6	11	14	14	10	3	52 (35.9)
Only if the patient initiates a conversation	14	10	5	2	12	6	8	5	-	31 (21.4)
Never	6	5	3	1	5	5	3	2	-	15 (10.3)
	How do you proceed if a patient undergoing VPA treatment has an unplanned pregnancy? Total, 145 participants who dispenced VPA during the past 12 months									
Participants, N	***45***	***49***	***35***	***16***	***36***	***39***	***35***	***30***	***5***	**145**
Advise the patient to urgently consult their prescribing physician	42	43	29	14	16	17	20	17	2	128 (88.3)
Counsel the patient regarding the teratogenic risk associated with VPA use during pregnancy	19	22	15	4	4	7	4	5	1	60 (41.4)
Counsel the patient to continue treatment until the next visit to a physician	11	9	12	2	9	10	6	8	1	34 (23.4)
	Other recommendations offered during patient counseling Total, 145 participants who dispenced VPA during the past 12 months									
Participants, N	***45***	***49***	***35***	***16***	***36***	***39***	***35***	***30***	***5***	**145**
Remind the patient about the need to periodically evaluate their treatment (at least annually)	30	40	25	11	26	29	26	23	2	106 (73.1)
Referred the patient to their treating physician because they were not using effective contraception	18	16	16	8	13	17	12	11	5	58 (40.0)
Counsel the patient to access the QR code embedded on the secondary package of VPA medicinal products	6	4	4	-	4	9	9	6	-	28 (19.3)
	What do you think would facilitate patient counseling and PPP adherence when it comes to pharmaceutical practice in Romania? Total, 144 participants who dispenced VPA during the past 12 months; optional question									
Participants, N	***45***	***49***	***34***	***16***	***36***	***39***	***34***	***30***	***5***	**144**

Availability of EMs in pharmacy	13	15	7	5	10	15	8	4	3	40 (27.8)
More time available to dedicate to patient counseling	10	9	6	2	10	5	6	6	-	27 (18.8)
Courses or training on using the EMs and the importance of counseling patients using VPA	14	8	10	6	12	7	7	11	1	38 (26.4)
Remuneration of counseling	5	16	9	2	2	10	11	8	1	32 (22.2)
	PARTIAL DISPENSING									
	Do you generally open the secondary package to partially dispense the medicine? Total, 145 participants who dispenced VPA during the past 12 months*									
Participants, N	***45***	***49***	***35***	***16***	***36***	***39***	***35***	***30***	***5***	**145**
Yes	39	39	29	13	33	30	29	23	5	120 (82.8)
No	6	10	6	3	3	9	6	7	-	25 (17.2)
	Do you offer the patient card or a copy of the patient information leaflet when partial dispensing is employed? Total, 120 participants, excluding the 25 participants who did not report partial dispensing*									
Participants, N	***39***	***39***	***29***	***13***	***33***	***30***	***16***	***13***	***3***	**120**
Yes	12	13	14	6	9	10	13	10	3	45 (37.5)
No	27	26	15	7	24	20	16	13	2	75 (62.5)

Abbreviations: EMs, educational materials; n, number of participants; PPP, pregnancy prevention programme; QR, quick response; VPA, valproate; Y/N, yes/no
