# Supplementary material for: Awareness of Risk Minimization Measures for Valproate and Pregnancy Prevention Program Compliance Among Pharmacists: A Cross-Sectional Survey in Romania
Source: Pharmaceuticals (Basel). 2025 Dec 5;18(12):1861. doi: 10.3390/ph18121861 (PMC12735724; doi:10.3390/ph18121861)
Supplement: Supplementary file 1 [file pharmaceuticals-18-01861-s001.zip › SupplMat02_BenchmarkTable_15nov25.pdf]

Supplementary Material 2

Table S3 Benchmark of VPA PPP implementation at community pharmacy level

Ref.	Study countries | Timeframe | Population	EM receipt	Card provision	Counselling	Key system levers
Current study, 2025	RO<br>Dec 2024-Feb 2025<br>Pharmacists (n, 267)	Receipt of information on PPP: 105 (39%)<br>**DHCP**<br>Receipt: 87/105 (83%)<br>Reading: 87/103 (84%)<br>**EMs**<br>Receipt: 74 (71%)<br>Reading: 74 (100%)	At every dispensing: 26 (23%)<br>**When partial dispensing (n, 120):**<br>Offer the patient card or a copy of the PIL: 45/120 (38%)	On teratogenic risk, at every dispensing: 65 (39%)<br>Emphasize the importance of effective contraception at every dispensing: 47 (32%)<br>**Unplanned prenancy:**<br>• Advise the patient to urgently consult their prescribing physician: 128 (88%)<br>• Counsel the patient on teratogenic risk: 60 (41%)<br>• Counsel the patient to continue treatment until the next visit to a physician: 34 (23%)	• Less than half of the participants actively received any type of information regarding the PPP<br>• Lack of availability of materials in pharmacy, low familiarity with the same, lack of dispensing system notification, remuneration of pharmaceutical service
Colas et al, 2024	FR, UK, SE, PL, DE, ES<br>Jul–Oct 2020<br>Pharmacists (n, 393)	**DHCP**<br>Receipt: 264 (67%)<br>Reading: 260 (66%)<br>**Guide for HCP**<br>Receipt: 188 (48%)<br>Reading: 211 (54%)<br>**Patient card**<br>Receipt: 213 (54%)<br>Reading: 194 (49%)	At every dispensing: 284 (72%)	**Advice on contraceptive methods to patients:** NA for pharmacists for this study<br>**When planning for pregnancy**<br>• Always remind or inform the patients about the risk of valproate in pregnancy: 244 (62%)<br>• Always refer the patient to her specialist: 311 (79%)<br>**Unplanned/suspected pregnancy**<br>• Urgently advise the patient regarding the timing to consult her valproate prescriber: 355 (90%)<br>• Recommend to continue treatment until the next visit with a physician: 311 (79%)	• Lower rates of receipt were reported for the patient card<br>• Contraception counselling and use of contraception in WCBP, whether or not they are sexually active

Spoge et al, 2024	BE, DK, GR, NL, PT, SK, ES, LV<br><br>Mar-Jul 2020<br><br>Pharmacists (n, 48)	-	Used/using it: 7 (14.6%)	Always inform/remind patients about the use of effective contraception: 21 (57%)<br><br>Always recommend stopping treatment if pregnancy is suspected: 14 (38%)<br><br>Always recommend contacting their doctor if pregnancy is suspected: 23 (62%)<br><br>Always emphasize the need for pregnancy tests before/during treatment: 9 (24%)	• A lack of confidence in the topic of contraception being adequately addressed and discussed<br>• While pharmacists actively emphasize the importance of contraception and recommend consulting a doctor if pregnancy is suspected, there is no consensus regarding the necessity of conducting pregnancy tests before or during treatment with VPA
Oliveri et al, 2023	DK<br><br>Jan 2019-Sep 2020<br><br>Pharmacists (n, 98)	-	At each dispensing: 5 (5.1%)	Always/often inform about effective contraception: 32 (33%)<br><br>Always/often stop treatment when pregnant: 18 (18%)<br><br>Always/often refer to prescriber when suspect a pregnancy: 38 (39%)<br><br>Always/often inform about pregnancy testing before/during treatment: 20 (20%)	• Providing information when dispensing VPA was found to be alarmingly low among Danish pharmacists, suggesting the need for a more prominent role of Danish pharmacists in the safe use of VPA, especially regarding pregnancy prevention.
Bertels et al, 2021	BE<br><br>Mar-Sep 2020<br><br>Pharmacists (n, 75)	-	-	Advises on the importance of contraception, always: 21%<br><br>Refers to a physician in case of a suspected pregnancy, always: 58%<br><br>*Note*: sample size: 62 pharmacists	• The results suggest inappropriate use of VPA in WCBP in BE

Hughes et al, 2021	IE<br><br>Jun-Aug 2019<br><br>Pharmacists (n, 96)	Received any PPP information: 79 (82.3%)<br><br>EMs as source of information: 37 (46.8%)	At each dispensing: 17 (17.7%)<br><br>**When broken bulk cannot be avoided, the following is offered**:<br><br>• Pharmacy warning sticker with pictogram: 23 (24.0%)<br>• Package leaflet/patient information leaflet: 53 (55.2%)<br>• Patient card: 41 (42.7%)	Counsel on the teratogenic risks at every dispensing: 10 (10.4%)<br><br>Reinforce the need for effective contraception at every dispensing: 18 (18.8%)<br><br>**Unplanned or suspected pregnancy when on VPA:**<br><br>• Advise the patient to contact the specialist managing her condition immediately: 95 (99%)<br>• Counsel the patient on the teratogenic risks: 82 (85%)<br>• Advise the patient to contact her GP immediately: 94 (98%)<br>• Advise the patient not to stop taking VPA unless advised to do so by her doctor: 89 (93%)	• Possible gaps in implementation of several key elements of the "prevent" PPP in current practice<br>• Implementation of elements of the PPP into community pharmacy practice varied; the focus appeared to be directed primarily towards new patients.

BE, Belgium; DE, Germany; DK, Denmark; EMs, educational materials; ES, Spain; FR, France; GR, Greece; IE, Ireland; LV, Latvia; NL, the Netherlands; PIL, patient information leaflet; PL, Poland; PPP, pregnancy prevention programme; PT, Portugal; RO, Romania; SE, Sweden; SK, Slovakia; UK, the United Kingdom; VPA, valproate; WCBP, women of childbearing potential

n, number of pharmacists

References:

Bertels X, Mehuys E, Boussery K, et al. The implementation of risk minimization measures to prevent teratogenic pregnancy outcomes related to oral retinoid and valproate use in Belgium. Acta Clin Belg. 2022 Oct;77(5):815-822. doi: 10.1080/17843286.2021.1983708. Epub 2021 Sep 26. PMID: 34569444.

Colas S, Nishikawa T, Dresco I, et al. Effectiveness of the Additional Risk Minimisation Measures for Valproate Among Healthcare Professionals and Patients: A Cross-Sectional Survey in Six European Countries. Pharmacoepidemiol Drug Saf. 2024 Nov;33(11):e70046. doi: 10.1002/pds.70046. PMID: 39532540.

Hughes JE, Buckley N, Looney Y, et al. (2021). Awareness, knowledge and practice of healthcare professionals following implementation of a Pregnancy Prevention Program for sodium valproate in Ireland: a multi-stakeholder cross-sectional study. Expert Opinion on Drug Safety, 20(8), 965–977. https://doi.org/10.1080/14740338.2021.1933429

Oliveri, N.M.B.; Hansen, J.M.; Almarsdóttir, A.B.; Jacobsen, R. The Awareness and Adherence of the Valproate Pregnancy Prevention Program: A Questionnaire Survey among Healthcare Professionals, Pharmacists, and Patients in Denmark. Int. J. Environ. Res. Public Health2023,20,2215. https:// doi.org/10.3390/ijerph20032215

Špoģe M, Kursīte M, Poplavska E. Latvian Healthcare Professionals' Self-Reported Knowledge, Attitudes, and Behaviors Related to Pregnancy Prevention Program Materials for Valproate-Containing Medicines. Pharmacy (Basel). 2024 Dec 4;12(6):182. doi: 10.3390/pharmacy12060182. PMID: 39728847; PMCID: PMC11679773.
