# Supplementary material for: Awareness of Risk Minimization Measures for Valproate and Pregnancy Prevention Program Compliance Among Pharmacists: A Cross-Sectional Survey in Romania
Source: Pharmaceuticals (Basel). 2025 Dec 5;18(12):1861. doi: 10.3390/ph18121861 (PMC12735724; doi:10.3390/ph18121861)
Supplement: Supplementary file 1 [file pharmaceuticals-18-01861-s001.zip › SupplMat03_Question_12nov25.pdf]

Supplementary Material 3

Table S4 **Questionnaire (EN translation)**

Eligibility

I confirm that I work as a pharmacist in an community pharmacy on the territory of Romania.

General Data 1.

1. How often have you dispensed medicinal products with valproic acid to women of childbearing age in the last 12 months?*

☐ Once or twice per week
☐ Twice per month
☐ Once per month or less
☐ Never

2. When did you learn about the teratogenic effects of valproic acid used during pregnancy?*

☐ During the past 2 years
☐ During the past 6 years
☐ More than 6 years ago
☐ Other <free text>

3. What sources were consulted regarding the safety profile of medicinal products with valproic acid?

☐ NAMMDR website
☐ EMA website
☐ I have not consulted any sources on this topic
☐ Other <free text>

Educational materials and direct communication receipt

4. Have you received any information related to the pregnancy prevention program (PPP)*

☐ Yes
☐ No <goes to #13>

5. Have you received the direct communication to healthcare professionals regarding the safety of valproic acid use during pregnancy?*

☐ Yes, via email
☐ Yes, printed
☐ No

6. Have you read the communication?*

☐ Yes, in full
☐ Yes, partially

☐ No

7. Have you received the educational materials regarding the safety of valproic acid use during pregnancy?*

☐ Yes
☐ No < goes to #13>

8. What were the educational materials received?

☐ Patient guide
☐ Patient card
☐ Risk acknowledgment form
☐ HCP guide

9. Were the educational materials received in a printed format or by email?

☐ Printed
☐ Email

10. Did you read the educational materials?*

☐ Yes, in full
☐ Yes, partially
☐ No

11. How useful for counseling patients did you find these materials?*

<1-5 scale>

12. Which educational material you find most useful for counseling patients?

☐ Patient guide
☐ Patient card
☐ Risk acknowledgment form
☐ HCP guide

13. Did you notice the QR code embedded on the secondary package?*

☐ Yes
☐ No

14. Did you know that by accessing the QR code you can view user information (e.g., guide, risk acknowledgement form, leaflet)?*

☐ Yes
☐ No

Pharmacists' compliance to PPP

The following questions relate to counseling women of childbearing age and the use of educational materials when dispensing valproic acid medications.

15. How often do you counsel the patient about the associated teratogenic risk?*

☐ Only in some cases
☐ At every dispensing
☐ Only if the patient initiates a conversation
☐ Never

16. How often do you emphasize the importance of effective contraception?*

☐ Only in some cases
☐ At every dispensing
☐ Only if the patient initiates a conversation
☐ Never

17. In the case of a patient undergoing treatment with valproic acid with an unplanned pregnancy, how do you proceed?

☐ I refer the patient to their treating doctor, urgently
☐ I counsel the patient regarding the teratogenic risk associated with the use of valproate during pregnancy
☐ I counsel the patient on the importance of not discontinuing valproate treatment unless instructed to do so by their doctor
☐ Other

18. What other recommendations do you offer to patients during counseling?

☐ I referred the patient to her GP because she was not using effective contraception.
☐ I reminded the patient of the need for periodic evaluation of the treatment (at least annually)
☐ I counselled the patient to scan the QR code on the product
☐ None of the above
☐ Other <free text>

19. How often do you use educational materials when dispensing these medications?*

☐ Only in some cases
☐ At every dispensing
☐ Never < goes to #24>

20. What materials do you use for counseling patients?

☐ Patient guide
☐ Patient card
☐ Risk acknowledgment form
☐ HCP guide
☐ Other <free text>

21. How often do you provide the patient with the card or recommend that the patient access the QR code on the secondary packaging for user information?*

☐ Only in some cases

☐ At every dispensing
☐ Never

22. Do you usually open the box for partial dispensing?*

☐ Yes
☐ No < goes to #25>

23. In these cases, do you provide a copy of the patient information leaflet or card?*

☐ Yes < goes to #25>
☐ No < goes to #25

24. What is the reason behind not using the educational materials for patient counseling?

☐ Not available in the pharmacy
☐ Not familiar with these materials
☐ I dont have them available close by
☐ I am not alerted by the dispensing software
☐ Other <free text>

25. What do you think could facilitate patient counseling at the pharmacy level and the implementation of the PPP for valproate in Romania?

☐ Availability of printed educational materials in the pharmacy
☐ More time available for patient counselling
☐ Trainings on the use of educational materials and the importance of patient counseling
☐ Remuneration of the pharmaceutical advisory service
☐ Other <free text>

How likely is it that the pharmacist will implement PPP and risk minimization measures in practice

26. In the future, how likely it is for you to consult the educational materials or direct communication for patient counseling?*

<1-5 scale>

27. Which of the following intervals define women of childbearing age in your opinion?

☐ 15-17 years
☐ 18-44 years
☐ 45-50 years
☐ 51-55 years
☐ Other <free text>

28. Do you have any other suggestions or aspects you would like to share regarding actual the implementation of the pregnancy prevention program? (optional)

<text>

Demographic data

Age group category (years)*

☐ ≤30
☐ 31-40
☐ 41-50
☐ >50

Sex*

☐ Female
☐ Male

Region*

☐ Urban
☐ Rural

Work experience (years)*

☐ 0-5
☐ 6-10
☐ 11-20
☐ 21-30
☐ >30

*unique response question
