# Supplementary material for: Awareness of Risk Minimization Measures for Valproate and Pregnancy Prevention Program Compliance Among Pharmacists: A Cross-Sectional Survey in Romania
Source: Pharmaceuticals (Basel). 2025 Dec 5;18(12):1861. doi: 10.3390/ph18121861 (PMC12735724; doi:10.3390/ph18121861)
Supplement: Supplementary file 1 [file pharmaceuticals-18-01861-s001.zip › SupplMat04_RIMES_12nov25.pdf]

Supplementary Material 4

Table S5 Reporting recommendations Intended for pharmaceutical risk Minimization Evaluation Studies-Standards for Reporting Implementaon Studies (StaRI) Extension: The RIMES-SE Checklist

Name of the study: Awareness of Risk Minimization Measures for Valproate and Pregnancy Prevention Programme Compliance Among Pharmacists: A Cross-Sectional Survey in Romania

Checklist Item		Reported on page #	Risk Minimization Program (Intervention)	Reported on page #	Program Implementation Strategy
**Title**	**1**	**1**	Identify as a risk minimization evaluation study, with description of the targeted safety risk, medicinal product, population, geographical location(s) and methodology in the title or keywords.		
**Abstract**	**2**	**1**	Identify as a risk minimization evaluation study, with a description of the risk minimization program including its goal(s) and theoretical basis (if known), how it was implemented and by whom, the target population, evaluation methods, and the key program process and outcomes.		
**Introduction**	**3**	**2**	Describe the problem, challenge, or deficiency in healthcare or public health that the risk minimization program aimed to address.		
	**4**	**Information unknown**	a. State the underpinning rationale (theory, model or framework, including any pilot work) guiding the design of the risk minimization program and how the program was expected to achieve its effects. (Report "None used "if no theory, model or framework used or report "Information unknown" if any of theabove information is not known.)	**Information unknown**	b. State the underpinning rationale (theory, model or framework, including any pilot work) for the implementation strategy/ies (theory, model or framework), and how it was expected to achieve its effects.) (Report "None used" if no theory, model or framework was used, or report "Information unknown" if any of the above information is not known.)
**Aims and Objectives**	**5**	**3**	Describe the aims of the risk minimization evaluation study, differentiating between program implementation and program impact.		
**Methods: Description**	**6**	**Not known**	Describe the context in which the risk minimization program was implemented (e.g., social, economic, healthcare, and/or organizational barriers and facilitators that might have influenced implementation).		
	**7**	**Not known**	a. Describe the population targeted by the risk minimization program and any eligibility criteria.	**Not known**	b. Describe the characteristics of the targeted healthcare sites for program implementation (type, location, personnel, etc.) and any eligibility criteria.
	**8**	**Not known**	a. Describe the risk minimization program, including the risk minimization measures, and whether any stakeholders were involved in the development.	**Not known**	b. Describe the risk minimization program implementation strategies. (Report "Unknown" if unknown whether or what type of implementation strategies were used.)
	**9**	**13-14**	a. Describe the outcome measures of the risk minimization program evaluation study (including knowledge-, behavior- and health-related), how assessed, and data sources, and the pre-determined thresholds of success. (Report	**13-14**	b. Describe the process measures prespecified as a goal of the evaluation study. (Report "Not assessed" or "Unknown" If not assessed or unknown.)

			“Unknown” if not no pre-specified threshold of success was specified.)		
	**10**	**Not assessed**	Describe the rationale for the study sample size (including effect size estimate, budgetary constraints, practical considerations, data saturation, as appropriate).		
	**11**	**13-14**	State the methods used for analysis (with reasons for that choice), including explanation of how missing data were handled.		
	**12**	**13-14**	Describe any planned sub-group analyses (e.g., between different sites, or different clinical or demographic populations).		
**Results**	**13**	**3**	Report proportion recruited and characteristics of the program population for the risk minimization evaluation study, including a table showing baseline characteristics of study sample and settings.		
	**14**	**8**	a. Report primary and other outcome(s) of the program evaluation, including whether primary outcome met a pre-specified success threshold, and statistic(s) for the precision of result estimate(s). (Report “Unknown” If pre-specified success threshold is unknown.)	**Not assessed**	b. State results of the analysis of the program implementation measures ("implementation outcomes”) associated with program implementation. (Report “Not assessed” if not assessed.)
	**15**	**Not assessed**	Describe any added time/procedures/processes incurred as a result of implementing the risk minimization program as compared to usual care (i.e., program ‘burden’).		
	**16**	**5-8**	Report the degree to which the risk minimization program measures were delivered completely and as intended to all participants (i.e., degree of ‘fidelity’) and description of any adaptations reported. (Report "Unknown" or "Not measured” if not measured.)		
	**17**	**Not assessed**	Report any contextual changes which may have affected program outcomes, including description of any factors that may have served to impede or facilitate program adoption.		
	**18**	**Suppl. material 1**	Report results of other analyses performed (e.g., important, pre-defined subgroup analyses, sensitivity analyses) and any evaluation of important harms and unintended effect(s) of the risk minimization program.		
**Discussion**	**19**	**8-14**	Summarize evaluation study findings, strengths and limitations, comparisons with other studies, conclusions and implications.		
	**20**	**Not assessed**	a. Discuss policy, practice and/or research implications of the risk minimization program (specifically including program sustainability).	**Not assessed**	b. Discuss policy, practice and/or research implications of the implementation strategy (specifically including scalability).
**General**	**21**	**14**	Include statement(s) on regulatory approvals (e.g., as appropriate, ethical approval, confidential use of routine data, governance approval), study registration (availability of protocol), funding and conflicts of interest.		

Reference: Smith MY, Morrato EH, Mora N, Nguyen V, Pinnock H, Winterstein AG. The Reporting Recommendations Intended for Pharma-ceutical Risk Minimization Evaluation Studies: Standards for Reporting of Implementation Studies Extension (RIMES-SE). Drug Saf. 2024;47(7):655-671
