# Supplementary material for: Awareness of Risk Minimization Measures for Valproate and Pregnancy Prevention Program Compliance Among Pharmacists: A Cross-Sectional Survey in Romania
Source: Pharmaceuticals (Basel). 2025 Dec 5;18(12):1861. doi: 10.3390/ph18121861 (PMC12735724; doi:10.3390/ph18121861)
Supplement: Supplementary file 1 [file pharmaceuticals-18-01861-s001.zip › SupplMat05_PPP_12nov25.pdf]

Supplementary Material 5

Table S6 PPP compliance items for pharmacists

Item	Participants : n/denominator
Awareness (n, 267 pharmacists)	
1. Receipt of DHPC (n, 105 responses)	87/105
2. Reading in full of the DHPC (n, 103 responses)	66/103
3. Receipt of EMs on safety of VPA during pregnancy (n, 105 responses)	74/105
4. Reading in full of the EMs (n, 74 responses)	54/74
5. Awareness of QR code leading to drug safety information and EMs (n, 267 responses)	130/267
Behaviour (n, 145 pharmacists)	
1. Counseled the patient on the teratogenic risk associated with VPA use during pregnancy at every dispensing. (n, 145 responses)	56/145
2. Outlined the importance of effective contraception at every dispensing. (n, 145 responses)	47/145
3. In case of unplanned pregnancy, advised the patient to urgently consult their prescribing physician at every dispensing. (n, 145 responses)	128/145
4. Counseled the patient to access the QR code embedded on the secondary package of VPA medicinal products/offered the patient card, at every dispensing (n, 111 responses, after excluding participants who never use EMs)	26/111
5. Generally, open the secondary package to partially dispense the medicine. (n, 145 responses); Correct responses were no or yes, but offered the patient card or a copy of the patient information leaflet in these cases.	25/145 (i.e., No)<br>45/145 (Yes + offer patient card/leaflet)

DHPC, direct healthcare professional communication; EM, educational materials; n, number of participants; QR, quick response; VPA, valproate
